# Psychosocial online counselling in Ukraine on IPSO-care platform in pandemic period

**DOI:** 10.1192/j.eurpsy.2021.1844

**Published:** 2021-08-13

**Authors:** V. Korostiy, I. Missmahl, O. Polishchuk, O. Platinuk

**Affiliations:** 1 Psychiatry, Narcology, Medical Psychology And Social Work, Kharkiv National Medical University, Kharkiv, Ukraine; 2 Main Office, International Psychosocial Organization, Kharkiv, Germany; 3 Medical Psychology Center, Bukovina State Medical University, Chernivtsi, Ukraine

**Keywords:** e-mental health, online counseling, psychotherapy, telepsychiatry

## Abstract

**Introduction:**

Since October 2017 till 2019 the project of Psychosocial Care for internally displaced persons and the war affected population in Ukraine has been in place (short name “Let’s talk”). In 2020 this project continuing for counselling pandemic affected persons.

**Objectives:**

The counsellors are professional psychologists who were trained within the scope of the project. Sessions may be held in Russian or Ukrainian, according to the user’s choice.

**Methods:**

Analysis of protocols of online counseling sessions and supervisions.

**Results:**

The key and most frequent issues mentioned by the clients are loneliness, the loss of the sense of life, fear, uncertainty, anxiety, difficulties in family relations, in particular, with children, job insecurity, addictions, psychosomatic disorders and so others. They are closely related to the situation in the country (military operation, the division into “We” and “Other”, the risk of provocations, the cases of treason and personal revenge, threats to the family members of the military). The above issue is in potential clients’ inadequately high suspicion level (in some cases on the border of paranoid fantasies), which is manifested in the fear of the possible infringement of confidentiality, over-listening, surveillance, recording of talks etc.

**Conclusions:**

The online counseling is the way to provide professional, accessible, free for the users and fully anonymous psychosocial care. Most frequent issues mentioned by the IDPs and the war affected population peoples has been indicated. When presenting the project service, the focus is always made on the high level of data protection and strict confidentiality.

**Disclosure:**

No significant relationships.

